# The prevalence of *optrA*-carrying *Enterococci* in the vaginal micro-ecology of pregnant women in late pregnancy

**DOI:** 10.1128/spectrum.02135-24

**Published:** 2024-11-29

**Authors:** Yanjun Xie, Fangyi Xu, Huali Dong, Jianfeng Mao, Chuanling Zhang

**Affiliations:** 1 Affiliated Xiaoshan Hospital, Hangzhou Normal University, Hangzhou, China; Chengdu University, Chengdu, Sichuan, China

**Keywords:** *Enterococci*, microenvironment, pathogenic microorganisms, oxazolidinone antibiotics, *optrA*, next-generation sequencing

## Abstract

The colonization of *Enterococcus* in the female vagina leads to neonatal and pediatric enterococcal septicemia. Linezolid (LZD) is a kind of mainstream drug for treating multidrug-resistant Gram-positive infections. *OptrA* is the main LZD-resistance gene at *Enterococci* in human isolates. It is essential to explore the prevalence of *optrA*-carrying *Enterococcus* in vaginal secretions of late pregnant women and the drug resistance of *optrA*. From May to June 2023, this study recruited 340 volunteers in late pregnancy (35–40 weeks of pregnancy) to provide non-repetitive vaginal discharge samples. Luria-Bertani broth and florfenicol (10 µg/mL) were used to enrich bacteria. *Enterococci* was identified through time-of-flight mass spectrometry. Additionally, antimicrobial susceptibility, polymerase chain reaction, and next-generation sequencing assays were applied to this study. Fifty-four *optrA*-carrying *Enterococcus* strains were obtained, the proportion of the whole vagina of late pregnant women was 15.88% (54 out of 340), and *Enterococcus faecalis* account the highest proportion. All *optrA*-carrying *Enterococcus* were resistant to at least three drugs. Tetracycline, chloramphenicol, erythromycin, and LZD have higher bacterial resistance rates. Genetic environment analysis revealed that IS*1216E*, *fexA*, and *erm*(A) may synergistically exert multidrug resistance with *optrA*. It is necessary to strengthen the surveillance of *optrA*-carrying *Enterococcus* in pregnant women. This study provides scientific support for controlling hospital infections and managing antibiotic-resistant bacteria, and provides a scientific basis for rational clinical medication.

**IMPORTANCE:**

The disruption of cervicovaginal microbiota homeostasis is considered a key factor in causing imbalance in the microenvironment, leading to inflammation, transmission of infections, and illness. *Enterococcus* is considered a major cause of healthcare-related infections globally. It has resistance to multiple antimicrobial drugs, which pose significant challenges for clinical treatment. Therefore, it is crucial to assess the prevalence of *optrA*-carrying *Enterococcus* in vaginal secretions of late pregnant women and the drug resistance of *optrA*. This study detected 15.88% of *optrA*-carried *Enterococci* in 340 pregnant women. Furthermore, we found that *optrA*-carrying *Enterococcus* strains are highly resistant to tetracycline, chloramphenicol, erythromycin, and Linezolid. Additionally, genetic environment analysis revealed that *IS1216E*, *fexA*, and *erm*(A) may synergistically exert multidrug resistance with *optrA*. This study provides scientific support for controlling hospital infections and managing antibiotic-resistant bacteria and provides a scientific basis for rational clinical medication.

## INTRODUCTION

The female reproductive tract colonized multiple microorganisms, and pathogenic microorganisms that were together with the host’s local immune and endocrine systems to maintain a stable microenvironment (1, 2). The disruption of cervicovaginal microbiota homeostasis is considered a key factor in causing imbalance in microenvironment, leading to inflammation, transmission of infections, and illness (3, 4). A Chinese report showed that Gram-positive cocci were the most common clinical pathogens in Jiangxi Child blood specimens (5). *Enterococci* are ubiquitous environmental Gram-positive bacteria and usually colonize the human and animal gastrointestinal tracts (6). It is considered a major cause of healthcare-related infections globally. In particular, *Enterococcus* faecalis and *Enterococcus* faecium have a strong survival ability and can adapt to the hospital environment (7). The *Enterococci* infection is one of the main pathogens for neonatal and child enterococcal septicemia (8). *Enterococcus* that colonizes the female reproductive tract may be the main source causing neonatal infection with *Enterococci* through the “fecal-vaginal-newborn” transmission route (9). Although neonatal sepsis treatment guidelines are available to guide the treatment of neonatal sepsis improving neonatal survival rate, the microbial antibiotic resistance limits the development of treatment strategies (10). Understanding the local epidemiology of drug-resistant *Enterococci* in late-gestation populations is beneficial in promoting the establishment of effective prevention and treatment strategies for neonatal sepsis.

*Enterococcus* has the characteristic of recruiting and exchange antibiotic resistance genes, and it has resistance to multiple antimicrobial drugs, including key antibiotics that serve as last-resort treatments, vancomycin, daptomycin, linezolid (LZD), and tigecycline, which pose significant challenges for clinical treatment (11). In cases where vancomycin cannot be used, severe enterococcal infections are usually treated with daptomycin, LZD, or combination therapy (6). LZD, the first oxazolidinone antibiotic used in clinical practice, is one of the main drugs for treating clinical infections caused by Gram-positive bacteria with multiple drug resistance (MDR) (12). However, emergence of LZD resistance is exacerbating the challenges of treating certain infectious diseases (13). So far, at least seven acquired LZD resistance genes have been reported, including Chloramphenicol-florfenicol resistance genes *cfr*, *cfr*(B), *cfr*(C), *cfr*(D), *cfr*(E), and ATP binding cassette (ABC) proteins of the F subtype (ABCF) genes *optrA* and *poxtA* (14–18). *Cfr* and its variants encode 23S rRNA methylase, *optrA* and *poxtA* encode an ABC-F protein (19). *OptrA* is the main gene for LZD-resistant *Enterococci* in human isolates (20). It can rapidly spread among different *Enterococcus* and other Gram-positive *cocci* with plasmids, transposons, etc. The existence and spread of resistance genes not only accelerate the acquisition of bacterial resistance, but also increase the difficulty of treating enterococcal-related infectious diseases. Therefore, this study aims to understand the resistance of late-pregnancy vaginal *optrA*-carrying *Enterococcus*, as well as its antimicrobial susceptibility, and its genetic environment*,* providing reference for rational drug use for vaginal *Enterococcus* infections in late-pregnant women and molecular epidemiological data for effective prevention and treatment of *Enterococcus* infections in pregnant women and newborns.

## MATERIALS AND METHODS

### Recruitment and sample collection

A total of 340 vaginal secretions were taken from 340 late pregnancy (35–40 weeks) pregnant women who underwent prenatal examinations in our obstetrics department from May to June 2023. A vaginal dilator was used to dilate the vagina, and a sterilized female swab was used to collect secretions from one-third of the vaginal sidewall or posterior fornix. All samples are processed and cultured within 1 h. All volunteers were free of urinary tract infections, intestinal infections, and other diseases. Antibiotic treatment was not administered 1 week before sampling, and only one strain of the same strain was selected from the same patient. This study was approved by the Ethics Committee of Zhejiang Xiaoshan Hospital (ethical clearance number: K2022035). Informed consent statements were obtained from all study participants.

### Main instruments and reagents

Bacterial genome extraction kits were purchased from Beijing TIANGEN BIOJING Co., Ltd. (Beijing, China). Luria-Bertani (LB) nutrient agar and LB broth medium were purchased from Beijing AOBOX Biotechnology Co., Ltd. (Beijing, China). Florfenicol, LZD, ciprofloxacin (CIP), vancomycin (VAN), tetracycline (TET), chloramphenicol (CPL), penicillin G, Na (PEN), and erythromycin (ERY) were purchased from Shanghai Aladdin Biochemical Technology Co., Ltd., (Shanghai, China). A PrimeSTAR Max DNA Polymerase kit (R045A), A DL1000 DNA Marker, and A MiniBEST Bacteria Genomic DNA Extraction Kit were purchased from Takara Biomedical Technology Co., Ltd. (Kyoto, Japan).

### Bacterial isolation and identification

Screening *Enterococcus* carrying *optrA* was referred to as an improved enrichment approach (21). To summarize, vaginal secretion samples were diluted using LB broth and inoculated at 37°C overnight. Then, 10 µg/mL florfenicol was added into LB broth containing 5% NaCl. Subsequently, samples were incubated at 37°C overnight. The sample dilution solution (20 µL) was spread on a blood agar plate (HuanKai Microbio, China) containing florfenicol (10 µg/mL) and was incubated at 37°C overnight, and initially screened for *Enterococci. Enterococci* was identified by MALDI-TOF MS (Burker, Saarbrucken, Germany) and repeated subculturing for pure culture.

### Antimicrobial susceptibility tests

Antimicrobial susceptibility testing performed using broth microdilution. *E. faecalis* (ATCC) strain was purchased from the Clinical Inspection Center of the National Health Commission of the People’s Republic of China and used as a quality control strain. The minimum inhibitory concentration (MIC) was determined using seven antimicrobial drugs: VAN, TET, LZD, CPL, PEN, CIP, and ERY. Resistant of strains was evaluated according to the American Clinical and Laboratory Standards Institute (CLSI) (22) criteria.

### Polymerase chain reaction assay

Polymerase chain reaction (PCR) assay was conducted to detect antibiotic-resistant genes. Primers of oxazolidinone resistance genes *optrA*, *poxtA*, *cfr*, and *cfr*(D) were sourced from prior studies (15, 23–25). Obtained through Beijing Tsingke Biotech Co., Ltd. (Beijing, China), these sequences are detailed in Table S1. Bacteria were collected from LB cultures, and then genomic DNA was isolated using a bacterial genomic DNA extraction kit (TIANGEN). PCR products were separated using electrophoresis of 1% agarose gel, stained with SYBR green, and imaged under a UV trans-illuminator. Positive results were sequenced at Beijing Tsingke Biotech Co., Ltd. followed by NCBI validation was performed for targeting gene alignment.

### Next-generation sequencing

Three bacteria stains (BD1017, BD1148, and BD1140) carrying *optrA*, *cfr,* and *poxtA* genes were subjected to next-generation sequencing (NGS), and sequencing data were assembled into contigs by SPAdes v.3.13.1 (26), and the sequences were annotated using the Rapid Annotations using Subsystems Technology (RAST) server (27) (http://rast.nmpdr.org). Subsequently, Basic Local Alignment Search Tool (BLAST), BLASTn and BLASTp (http://blast.ncbi.nlm.nih.gov/blast) were used for sequence comparison analysis. Antagonistic genes were identified using the Center for Genomic Epidemiology (CGE) server (https://cge.cbs.dtu.dk/services/). Bacterial insertion sequences (ISs) were annotated using ISFinder (28) (https://isfinder.biotoul.fr/). Comparative analysis and mapping were performed using Easyfig v2.2.2 (29) (https://mjsull.github.io/Easyfig/).

## RESULTS

### Isolation of *optrA*-carrying *Enterococci*

**Fig 1:**
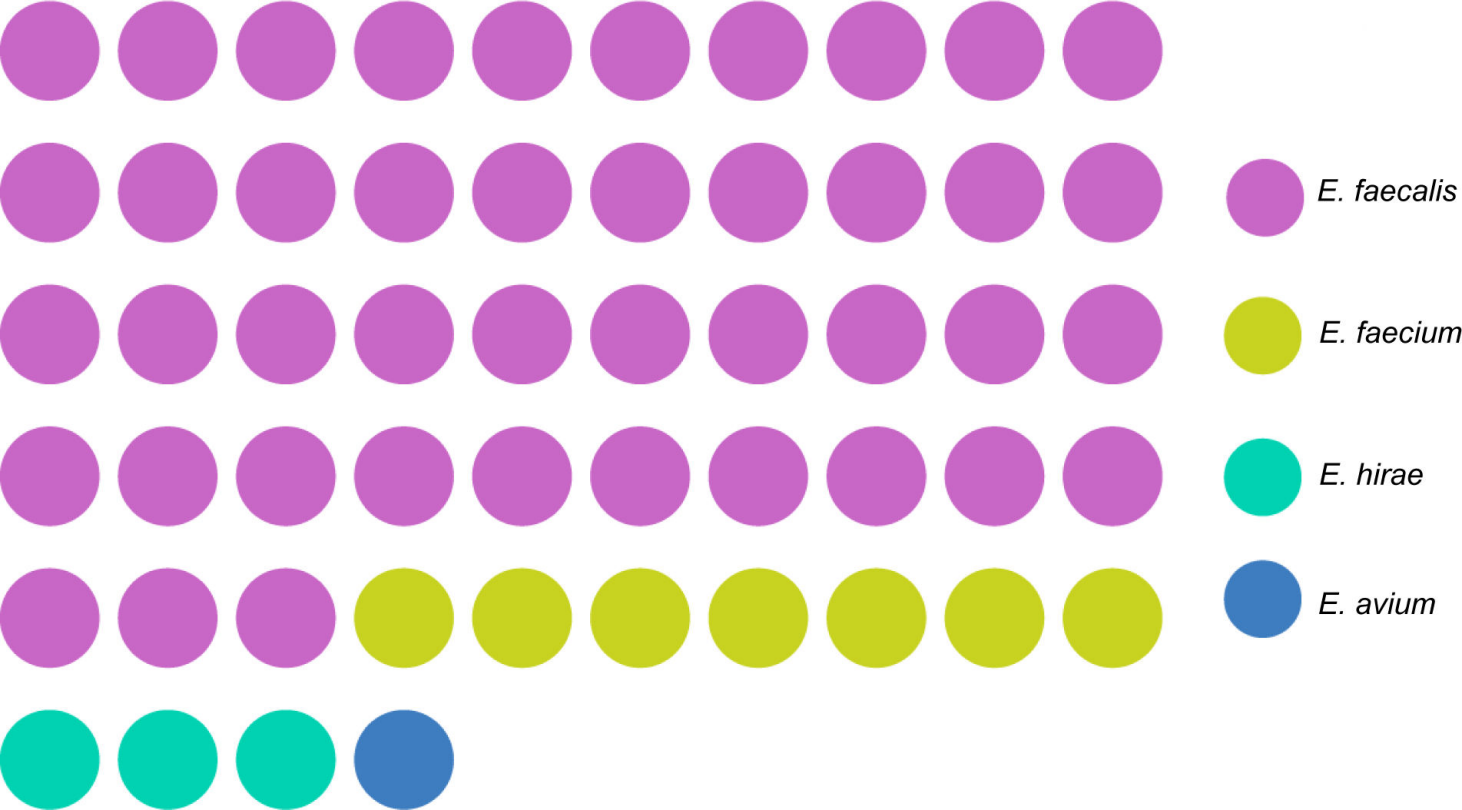
Distribution of *optrA*-carrying *Eenterococci*. Colored circles were used to represent each strain, totaling 54 strains. Forty-three pink circles represent *Enterococcus faecalis* (*E. faecalis*), seven yellow circles represent *Enterococcus faecium* (*E. faecium*), three green circles represent *Eenterococcus hirae* (*E. hirae*), and one blue circle represents *Enterococcus avium* (*E. avium*).

Using a culture medium containing florfenicol and MALDI-TOF MS mass spectrometry isolated *optrA*-carrying Gram-positive bacteria (21). As shown in Fig. 1, 54 *Enterococcus* strains were isolated from 340 vaginal secretion samples at an isolation rate of 15.88%. The identified species of *Enterococcus* included 43 *E. faecalis*, 7 *E. faecium*, 3 *Enterococcus hirae*, and 1 *Enterococcus avium*. Among them, *E. faecalis* was the main epidemic, followed by *E. faecalis*. Additionally, *Enterococcus*-positive group presented 18 cesarean sections and 35 vaginal deliveries (Table S2). A non-significant chi square test result was observed when compared to the negative control.

### Identification of drug resistance genes in *Enterococci*

**Fig 2:**
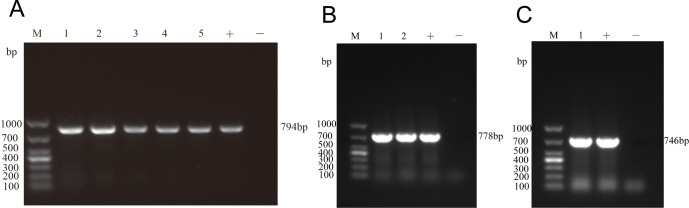
Representative PCR gel picture of *optrA*, *poxtA*, and *cfr* from *optrA*-carrying *Eenterococci*. (**A**) PCR results of *optrA*. M: DL1000 DNA Marker; 1, 2, 3: Strain sample; +: Positive control; -: Negative control; (**B**) PCR results of *poxtA*. M: DL1000 DNA Marker; 1, 2, 3: Strain sample; +: Positive control; -: Negative control; and (**C**) PCR results of *cfr*. M: DL1000 DNA Marker; 1: Strain sample; +: Positive control; -: Negative control.

PCR results showed that all *Enterococcus* (54 strains) carried the *optrA* gene, of which two strains (BD1017 and BD1148) carried both *optrA* and *poxtA* genes and were identified as *E. faecalis*, and one strain (BD1140) carried both *optrA* and *cfr* genes and was identified as *E. hirae*. The results of agarose gel electrophoresis are shown in Fig. 2A through C, and the homology rate between the measured sequences and the target gene sequences was over 99%, which was in line with expectations.

### Antibiotic sensitivity of *optrA*-carrying *Enterococci*

According to the CLSI drug sensitivity folding point judgment criteria (22), 54 *optrA*-positive *Enterococci* strains have different degrees of antibiotic sensitivity to seven drugs (VAN, TET, LZD, CPL, PEN, CIP, and ERY) (Table 1). The highest resistance rates were 98.1% (53 out of 54) and 94.4% (51 out of 54) for TET and CPL, followed is ERY with a resistance rate of 90.7% (49 out of 54). CIP and PEN had lower resistance rates of 29.6% (15 out of 54) and 12.9% (7 out of 54), respectively. No *Enterococcus* shows the resistance to VAN. Notably, 35 strains were resistant to LZD (MIC ≥8 µg/mL), with a resistance rate of 64.8%.

**TABLE 1:** Resistance phenotypes of 54 *optrA* positive strains

Antibiotics	MICs^*a*^^a^ (mg/mL)											MIC_50_ (μg/mL)	MIC_90_ (μg/mL)	Drug resistance rate
	0.25	0.5	1	2	4	8	16	32	64	128	256			
VAN^*a*^	4	28	21	0	1	0	0	0	0	0	0	0.5	1	0%
TET^*a*^	0	1	0	0	0	0	0	36	17	0	0	32	64	98.1%
LZD^*a*^	0	0	1	7	11	33	2	0	0	0	0	8	8	64.8%
CPL^*a*^	0	0	0	0	0	0	3	20	27	4	0	64	64	94.4%
PEN^*a*^	0	1	1	2	24	19	2	0	5	0	0	4	16	12.9%
CIP^*a*^	0	1	33	4	1	15	0	0	0	0	0	1	8	29.6%
ERY^*a*^	0	0	0	1	4	2	0	5	42	0	0	64	16	90.7%

^*a*^ VAN: Vancomycin; TET: tetracycline; LZD: Linezolid; CPL: Chloramphenicol; PEN: Penicillin; CIP: Ciprofloxacin; ERY: Erythromycin. The brown area represents the resistant area; MIC: minimum inhibitory concentration.

### Resistance profiles of *optrA*-carrying *Enterococci*

Fifty-four optrA-positive strains were resistant to more than three antimicrobial drugs, with a total of nine resistance profiles (Table 2), showing multidrug resistance. Twenty-three strains resistant to three antibiotics with three profiles; 15 strains resistant to four antibiotics with three profiles; and 14 strains resistant to five antibiotics. Notably, two strains were resistant to six antimicrobials. Three strains (BD1017, BD1140, and BD11148) carrying two oxazolidinone resistance genes were also resistant to four or more drugs, showing high resistance.

**TABLE 2:** Resistance profiles of 54 *optrA*-carrying *Enterococcus* strains

Isolates number	Antimicrobials number	Resistance patterns^*a*^
3	3	TET + CIP + ERY
5	3	TET + LZD + CPL
15	3	TET + CPL + ERY
1	4	LZD + CPL + PEN + ERY
1	4	TET + CPL + CIP + ERY
13	4	TET + LZD + CPL + ERY
4	5	TET + LZD + CPL + PEN + ERY
10	5	TET + LZD + CPL + CIP + ERY
2	6	TET + LZD + CPL + CIP + PEN + ERY

^*a*^ VAN: Vancomycin; TET: tetracycline; LZD: Linezolid; CPL: Chloramphenicol; PEN: Penicillin; CIP: Ciprofloxacin; ERY: Erythromycin.

### Genetic environment analysis of *optrA*

**Fig 3:**
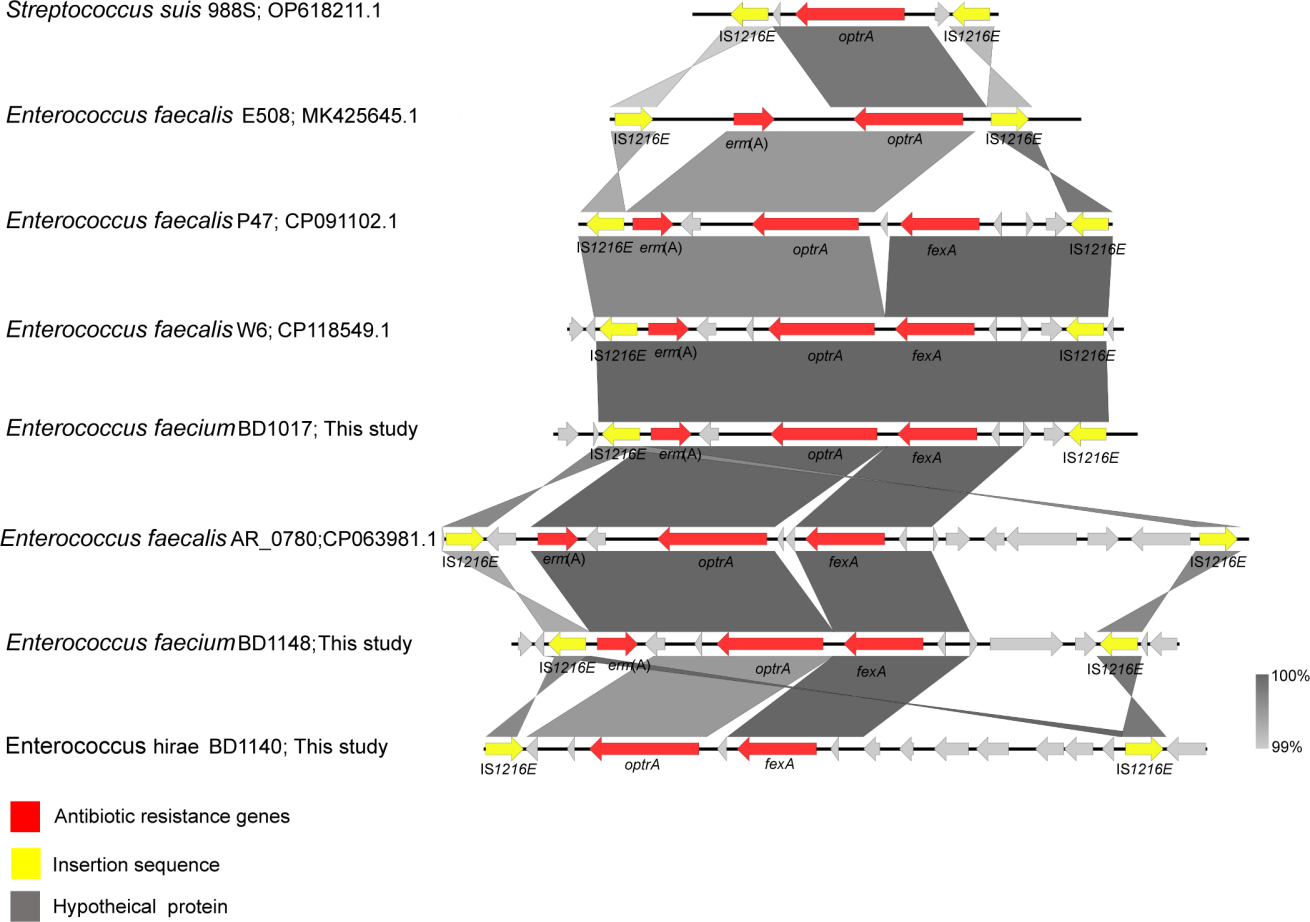
Genetic environment analysis of *optrA* gene in *Eenterococci*. Red represents antibiotic resistance genes, yellow represents insertion sequence, and gray represents hypothetical proteins.

The NGS results of three *Enterococcus* (BD1017, BD1140, and BD1148) were compared and analyzed. As shown in Fig. 3, *erm*(A) and IS*1216E* genes are upstream of *optrA* gene in BD1017 and BD1148 strains, and *fexA* and IS*1216E* are downstream of *optrA* gene, which formed an IS*1216E-erm*(A)-*optrA-fexA*-IS*1216E* transposable unit. In BD1140, the *optrA* gene is linked to *fexA* gene and its upstream and downstream were both isotropic IS*1216E* gene, forming a transposable unit of IS*1216E-optrA-fexA*-IS*1216E*. Compared to the genetic environment of *optrA* in the NCBI database, the presence of IS*1216E* promoted evolved strains and carried more drug-resistant genes, resulting in the risk of drug-resistant gene transmission.

## DISCUSSION

LZD is an antimicrobial drug approved by the US Food and Drug Administration (FAD) for clinical use in human medicine in 2000 for the treatment of infections caused by Gram-positive cocci infections (30). *OptrA*-mediated resistance to LZD is on the rise in Gram-positive organisms, significantly limiting treatment options for serious bacterial infections. In 2015, Cai et al. (31) investigated fecal carriage of *optrA*-positive *E. faecalis* from an asymptomatic, healthy population in Hangzhou, China, and found that *optrA* carriage rates were about 3% in adult samples (55 out of 1, 558) and about 3.5% in children (66 out of 1,900). In 2016–2017, a report from Jiangsu, China, showed that (32) LZD resistance rate against *Streptococcus suis* isolates was 38.3%. Furthermore, in 2022, Shen et al. (21) screened fecal samples from 1,018 healthy donors in Hangzhou, China, and the total *optrA* positivity rate of *optrA*-positive bacteria was 22.50% (256 out of 1,018). In 2024, Wang et al. (33) reported that *optrA* is the most common gene for LZD resistance. Although there is a lack of epidemiological analysis over a longer period, the above studies still support the view that LZD resistance needs to be taken seriously. The emergence of LZD-resistant bacteria in certain geographic regions necessitates further research and monitoring.

In this study, 54 strains of *Enterococcus* were isolated from 340 vaginal secretion samples of late-gestation volunteers. We found that the positive rate of carrying *optrA* gene was 100%, suggesting that we successfully isolated *optrA*-carrying Gram-positive bacteria using a blood agar plate containing florfenicol. This supports Shen et al.’s report (21). Furthermore, we found that two strains carrying *poxtA* and *optrA* genes, and one strain carrying *cfr* and *optrA* genes, and no strains carrying *cfr*(D) gene were detected, suggesting that the resistance mechanism of *cfr*(D) gene may be independent of *optrA*. Although studies have explored the potential mechanism of *optrA* resistance, evidence suggests that *optrA* resistance to LZD does not involve active efflux (34). There is still a lack of understanding about *optrA* mediated resistance to LZD, which deserves more in-depth and comprehensive research.

Therefore, we analyzed the genetic environment of *optrA* in *optrA*-carrying Gram-positive bacteria. We found that both upstream and downstream of *optrA* were engaged by the IS*1216E* for circular transposons, resulting in *optrA* transmission. Furthermore, *optrA* is part of a transposable element that forms a complex with *fexA* and *erm*(A). It has been revealed that *optrA*-carrying strains may cause not only florfenicol/LZD resistance, but also erythromycin resistance. Consequently, *optrA* dissemination could potentiate multidrug resistance in conjunction with other resistance genes. Our findings underscore the importance of prompt patient surveillance, prevention, and control measures, coupled with judicious medication usage.

*Enterococci* can cause not only urinary tract infections and soft tissue skin infections, but also life-threatening abdominal infections, sepsis, endocarditis, and meningitis (35, 36). Notably, late-stage pregnant women carrying this bacterium could contribute to neonatal sepsis, especially among newborns and young children. There is a six-fold increase in the incidence of enterococcal sepsis in newborns and children (8). The high prevalence of *Enterococcus* strains in *optrA-carrying* Gram-positive indicates that *Enterococcus* could potentially be the driver of *optrA* transmission. Reasonable management of *Enterococcus* infection may effectively manage the increase in LZD resistance. There is a need for further routine and ongoing screening of pregnant women in late pregnancy for risk factors to prevent the rapid spread of LZD resistance.

This study provides a reference for managing *Enterococcus* infections in late-pregnant women, but the potential limitations of conclusions due to limited sample size and duration of sampling are noted. Therefore, we plan to conduct comprehensive and enduring observations in the future to support similar studies.

### Conclusion

In summary, in this study, 15.88% of *optrA*-carried *Enterococci* were detected in pregnant women from our hospital, and *Enterococcu*s was identified as a combination of *optrA* and other resistant genes. And *optrA*-carried *Enterococci* are resistant to TET, LZD, CPL, PEN, CIP, and ERY. Genetic environment analysis revealed that IS*1216E*, *fexA*, and *erm*(A) may synergistically exert multidrug resistance with *optrA*. This study provides scientific support for controlling hospital infections and managing antibiotic-resistant bacteria and provides a scientific basis for rational clinical medication.

## SUPPLEMENTAL MATERIAL

Supplemental material spectrum.02135-24-s0001.docx Tables S1 and S2.

ASM does not own the copyrights to Supplemental Material that may be linked to, or accessed through, an article. The authors have granted ASM a non-exclusive, world-wide license to publish the Supplemental Material files. Please contact the corresponding author directly for reuse.
