# Supplementary material for: The prevalence of optrA-carrying Enterococci in the vaginal micro-ecology of pregnant women in late pregnancy
Source: Microbiol Spectr. 2024 Nov 29;13(1):e02135-24. doi: 10.1128/spectrum.02135-24 (PMC11705934; doi:10.1128/spectrum.02135-24)
Supplement: Supplemental material — Tables S1 and S2. [file spectrum.02135-24-s0001.docx]

Supplementary files

Table S1 Primer sequences for each gene

| Gene | Primer sequence (5’-3’) | Annealing temperature (°C) | Amplification length（bp） |
| --- | --- | --- | --- |
| *optrA* | F: GCACCAGACCAATACGATACAA  R:TCCTTCTTAACCTTCTCCTTCTCA | 58 | 794 |
| *poxtA* | F: GGTCTGACTGGCTTGTTTTGCT  R: ATAAGGTCGGTATTGTCGGCGT | 60 | 778 |
| *cfr* | F: TAAGAAGTAATAATGAGC  R: TATAGAAAGTCTACGAGG | 45.3 | 746 |
| *cfr(D)* | F: TGCGCTACTGGAAAAATTGGC  R: GCTTGAACGTTCTTGGTGCAT | 57 | 556 |

Table S2 Gestational outcomes of *Enterococci*-positive pregnant women

|  | Negative control | *Enterococcus* positive | *P^1)^* |
| --- | --- | --- | --- |
| Cesarean section | 25 | 18 | 0.323 |
| Vaginal delivery | 33 | 35 |  |
| Total | 58^2)^ | 53^3)^ |  |

1) Chi-Square Tests

2) Total of the negative control group are 286 cases and 228 of the gestational outcomes were lost.

3) Total of the *Enterococcus* positive group are 54 cases and one of the gestational outcomes was lost.
